# Health care utilization for acute illnesses in an urban setting with a refugee population in Nairobi, Kenya: a cross-sectional survey

**DOI:** 10.1186/1472-6963-14-200

**Published:** 2014-05-02

**Authors:** Abdinoor Haji Mohamed, Warren Dalal, Raymond Nyoka, Heather Burke, Jamal Ahmed, Erick Auko, Wilbert Shihaji, Irene Ndege, Robert F Breiman, Rachel B Eidex

**Affiliations:** 1 Kenya Medical Research Institute, Mbagathi road off Mbagathi way, KEMRI main campus, CDC-Building, Nairobi, Kenya; 2 US Centers for Disease Control and Prevention, Division of Global Migration and Quarantine, Atlanta, GA, USA; 3 US Centers for Disease Control and Prevention, Refugee Health Program for Africa, Nairobi, Kenya; 4 International Organization for Migration, Nairobi, Kenya

**Keywords:** Health care utilization, Urban refugees, Eastleigh, Kenya

## Abstract

**Background:**

Estimates place the number of refugees in Nairobi over 100,000. The constant movement of refugees between countries of origin, refugee camps, and Nairobi poses risk of introduction and transmission of communicable diseases into Kenya. We assessed the care-seeking behavior of residents of Eastleigh, a neighborhood in Nairobi with urban refugees.

**Methods:**

During July and August 2010, we conducted a Health Utilization Survey in Section II of Eastleigh. We used a multistage random cluster sampling design to identify households for interview. A standard questionnaire on the household demographics, water and sanitation was administered to household caretakers. Separate questionnaires were administered to household members who had one or more of the illnesses of interest.

**Results:**

Of 785 households targeted for interview, data were obtained from 673 (85.7%) households with 3,005 residents. Of the surveyed respondents, 290 (9.7%) individuals reported acute respiratory illness (ARI) in the previous 12 months, 222 (7.4%) reported fever in the preceding 2 weeks, and 54 (1.8%) reported having diarrhea in the 30 days prior to the survey. Children <5 years old had the highest frequency of all the illnesses surveyed: 17.1% (95% CI 12.2-21.9) reported ARI, 10.0% (95% CI 6.2-13.8) reported fever, and 6.9% (3.8-10.0) reported diarrhea during the time periods specified for each syndrome. Twenty-nine [7.5% (95% CI 4.3-10.7)] hospitalizations were reported among all age groups of those who sought care. Among participants who reported ≥1 illness, 330 (77.0%) sought some form of health care; most (174 [59.8%]) sought health care services from private health care providers. Fifty-five (18.9%) participants seeking healthcare services visited a pharmacy. Few residents of Eastleigh (38 [13.1%]) sought care at government-run facilities, and 24 (8.2%) sought care from a relative, a religious leader, or a health volunteer. Of those who did not seek any health care services (99 [23.0%]), the primary reason was cost (44.8%), followed by belief that the person was not sick enough (34.6%).

**Conclusion:**

Health care utilization in Eastleigh is high; however, a large proportion of residents opt to seek care at private clinics or pharmacies, despite the availability of accessible government-provided health care services in this area.

## Background

In 1996, almost all refugees under the protection of the United Nations High Commissioner for Refugees (UNHCR) around the world lived in camps [1]. However, UNHCR’s most recent report indicates that almost half of the world’s 10.5 million refugees now live in cities and towns, compared with one third who live in camps and one third who live in rural or dispersed areas [2]. As of June 2012, the total official refugee population in Kenya stood at 623,873, of which 471,110 (75.5%) live in Dadaab refugee camps, 98,380 (15.8%) live in Kakuma refugee camp, and 54,383 (8.7%) live in Nairobi, with 300 to 500 new refugees arriving in Nairobi every week [2,3]. The total numbers of officially registered urban refugees are lower than seen elsewhere in the world, mainly due to Kenyan government policies requiring refugees to live in designated refugee camps [4,5]. Unofficial estimates place the number of refugees in Nairobi at 100,000 to 150,000 [6]. Refugees moving to Nairobi and other towns in Kenya are motivated to improve their economic conditions outside the camp situation [7]. The urban refugee population is highly mobile and reluctant to come forward due to immigration laws and encampment policies in Kenya. Despite the large presence of refugees in urban centers, health programs and other interventions by national and international organizations still focus to a large extent on camp-based refugees [8].

Urban refugees in Kenya originate from a number of neighboring countries. Most urban refugees from Somalia, Ethiopia, and Eritrea live in and around the neighborhood of Eastleigh [2], a suburb in eastern Nairobi inhabited largely by Somalis. The Somali population in Eastleigh includes both Kenyan nationals and people who have migrated from Somalia [9]. The presence of this large Somali community in Eastleigh attracts new refugee arrivals from Somalia as well as from the two camp sites, Dadaab and Kakuma. Part of the draw to settle in Eastleigh is the hope of finding better job opportunities and education [2,9]. The constant multidirectional movement between countries of origin, refugee camps, and the Eastleigh area poses risk of introduction and transmission of communicable diseases in the region and beyond the borders of Kenya. In late 2005, the index case for a measles outbreak in Eastleigh traveled to a refugee camp in northern Kenya and back to Nairobi, resulting in the spread of measles in other parts of Kenya and eventually to other countries like the United States, Netherlands, Canada, and Mexico [10]. The combination of inadequacies in the surveillance of communicable diseases and limited diagnostic capacity at the public health care facilities presents challenges in early detection and control of outbreak-prone infectious diseases in this area [2].

Health care in Kenya is provided by government and nongovernment organizations, including Kenya’s ministry of health and local government authorities, religious organizations and private for-profit health care providers. The private health care system in Kenya has grown over the last two decades; among the reasons for this growth are lack of adequate and quality public health care services and introduction of user fees. The quality of services in this continuously growing private health sector is not regulated. This growth can also be associated with the health sector reforms undertaken in the 1980s and 1990s, when the government relaxed the licensing and regulation of private health care providers and the prohibition of public sector personnel from working in private sector [11]. The mobility of Eastleigh residents, coupled with the limited disease surveillance activities and the presence of unregulated health care providers, makes information on health-seeking behavior in Eastleigh essential for targeting public health prevention and disease detection programs.

Respiratory, febrile, and diarrheal diseases are among the leading causes of morbidity in Kenya [12]. Health care utilization surveys are used to provide information on the burden of important infectious diseases in populations and to plan improved disease (and epidemic) detection surveillance systems through knowledge of where residents tend to seek care. We conducted a cross-sectional survey to assess utilization of health care services for episodes of acute illness in children and adults in Eastleigh, a neighborhood in Nairobi with a large urban refugee population, to better understand the health needs of a community that is often difficult to access.

## Methods

### Setting and population

We conducted a health care utilization survey (HUS) in Eastleigh North Division, Kamukunji district, Nairobi Province, Kenya. Eastleigh North, one of five divisions in the district, occupies an area of 0.88 Km^2^. During July and August 2010, we sampled participants living in an area of Eastleigh North Division called Section II, which has a large number of multiple dwelling settlements and single-room occupancy units as well as a large number of small shops. All households in the selected area and all persons living in the selected households were considered eligible. For the purposes of the survey, a household was defined as people who slept in the same compound and shared the same cooking arrangements. An individual was considered a member of a selected household if he/she slept within a compound, apartment, or room within the study area for at least 3 of any of the preceding 12 months. Infants were included from the time of birth if their mother fit the eligibility requirements. Individuals who were part of the household but had died within the preceding 12 months were included as well, if they had an episode of one of the illnesses of interest and met the inclusion criteria.

For the purposes of the survey, we defined a caretaker as a member of the household who resided in the survey area for at least three of the past 12 months, was at least 18 years of age or older, and was the person identified as being responsible for the health of the members of the household. Selected households in which the caretaker did not consent to participate were not surveyed. Persons living in hotels or other facilities that rent rooms by the night and visitors who stayed in the household less than 3 months in the preceding 12-month period were excluded.

### Sampling

We used a multistage cluster sampling design [13]. In the first stage, we randomly identified blocks (areas demarcated by four streets including several apartment buildings and residential compounds) in Section II of Eastleigh North. In the second stage, we used probability proportional to size (PPS) to select plots, since the distribution of households in the plots was not uniform (plots were defined as apartments or compounds inside blocks that contained several households) within the selected blocks. In the third stage we randomly identified households within the selected plots to participate in the survey. We used the standard sample size calculation formula for cluster sampling in cross-sectional studies and adjusted for a design effect of two and an inflation factor of 0.85 [14], resulting in a targeted sample size of 785 households.

### Health utilization survey

Data were gathered by interview teams over a 3-week period from July 29, 2010, through August 14, 2010. The survey followed a 3-week training of 21 community interviewers, 1 week of mapping the selected households, and a week of piloting the survey instrument. Survey teams were grouped into three teams that included six community interviewers, two community mobilizers, two guides, and one team leader. Appointments for interviews with caretakers were arranged in advance by community mobilizers, who were community leaders well known in the area. Young adults from the community guided the community interviewers to the households where the mobilizers had visited and set an appointment.

If the caretaker or other household member was not present at the time of the scheduled interview, the community interviewers made three additional attempts to include the household in the survey. Single replacement households were randomly selected in advance and were used as substitutes for households that were not available for the survey, refused, or did not meet eligibility requirements. Written consent to participate in the HUS was obtained from all interviewees. The questionnaires were translated and administered in both English and Somali languages to participating caretakers; however, if a caretaker spoke another language an interpreter was provided. Caretakers served as proxies to answer questions for children less than 18 years of age or for people unavailable at the time of the interview.

The survey was organized into four parts. The first part focused on household demographics, water and sanitation. The second part identified and screened household members for illnesses of interest. The third part involved collecting information relating to the illnesses that occurred in the household, and the fourth part focused on utilization of health care services for that illness. To ascertain use of health care services, we specifically asked if health care was sought; by what type of provider and why the individual sought or did not seek care. The duration of the survey varied between 15 to 60 minutes per household, depending on the number of people in the household and the number of recent illnesses.

Standard case definitions were developed to assess the illnesses that occurred at the household level. *Fever* was defined as an illness associated with feeling hot or feverish during the 2 weeks prior to the interview. *Diarrhea* was defined as three or more loose stools over a 24-hour period during the month prior to the survey. *Acute respiratory illness* (ARI) was defined as either an illness with cough and difficulty in breathing for longer than 2 days or a diagnosis of “pneumonia” by a health care worker such as a doctor, clinical officer, or a nurse during the year prior to the survey. Three detailed questionnaires related to each of the three illness categories.

If a household had multiple members with the syndromes of interest older than 5 years, one child (5–17 years) and one adult (≥18 years) were interviewed regarding their illness. If there was someone who died of the illness in the age group that person was selected, otherwise, the person most recently ill was chosen and If 2 people were ill at the same time, the older one was picked. All children <5 years whose illness fit the case definitions were included, regardless of the number in a household. The same questions concerning health care-seeking behavior were asked for each syndrome. When multiple instances of a syndrome occurred within the relevant time frame, the questionnaire asked only about the most recent episode of the illness. A maximum of two detailed illness-specific questionnaires was administered for any one individual. Diarrhea or respiratory symptoms are often accompanied by fever, making it difficult for respondents to differentiate the associated symptoms; so when fever was reported with either diarrhea or acute respiratory infection, we administered only the detailed questionnaire related to the diarrhea or acute respiratory infection symptoms. If both diarrhea and pneumonia were reported, questions relevant to both syndromes were asked.

### Analysis

Data were double entered into a Microsoft Access 2007 database (Microsoft Access; Microsoft, Redmond, WA). After data cleaning, all analyses were conducted by using SAS software version 9.2 (SAS Institute Inc., Cary, North Carolina, USA).

The analysis was carried out by taking into account clustering in the survey design. We used sampling weights to compensate for unequal probabilities of selection [15]. In our survey, which was a multistage sample design, the probability of selection is the joint probability of selection at the three stages of sample selection. The sampling weight assigned to a household in a given cluster is the inverse of its probability of selection. Weights were standardized to avoid generating incorrect standard errors and confidence intervals [15,16]. Statistical significance was considered to be reached if p value was <0.05 in the logistic regression model, however, in the multivariate analysis variables with p value <0.1 were included.

To group households according to socioeconomic status (SES), data were gathered on household possessions and other variables associated with economic correlations (ownership of a conventional television, digital satellite television, radio, refrigerator, source of drinking water, availability of treated water, presence of household toilet, and availability of soap for washing hands). All categorical variables were converted into binary categories and then fitted into a Principal Component Analysis (PCA) model [17]. Variables of household possessions with more unequal distribution were given more weight in the PCA [17]. Households with at least one missing value, 16 (16%), were excluded from the analysis. The first principal component was used to classify households into SES categories. We classified the households using quintiles as cut-off points. The top quintile was classified as high SES, the second and third quintiles as middle SES, and the last two quintiles as low SES. Households who ranked in the top 20% with regard to SES variables were put in the higher socioeconomic group, the middle 40% in the middle socioeconomic group, and the lowest 40% in the lower socioeconomic group [17-19] .

To stratify illness episodes into severe and non severe categories we used a simple count of the symptoms reported by the participants (i.e., the sick person or the caretaker). We defined a minimum number of symptoms for each illness of interest; for respiratory illnesses to be considered severe the household member must have experienced a minimum of four symptoms (these symptoms included; fever or temperature >38°C, chills, cough, difficulty in breathing, fast breathing, chest indrawing, nasal flaring, wheezing, breathing with grunting noises, blue mouth and/or fingers, unconsciousness, convulsions, chest pain with breathing and cough with blood,). Persons categorized as having had severe diarrheal illnesses must have experienced a minimum of four symptoms(these symptoms included; 3 or more loose or watery stools in a day, vomiting, irritability or restlessness, decreased activity or lethargy, loss of consciousness, fever/chills, nausea and blood in stool), and those categorized as having severe febrile illness must have experienced a minimum of three symptoms(this symptoms included; fever or temperature >38°C, chills, convulsions, vomiting, decreased activity or lethargy, loss of consciousness, cough, difficult/fast breathing, joint pain, muscle pain/body pains, abdominal pain, headache, bleeding from nose/mouth/other orifice, diarrhea, rash, ear or throat pain, skin infection and difficulty or pain when urinating) [20].

To determine whether factors influenced health care-seeking behaviors among people with different countries of origin, we stratified the data based on country of origin and analyzed each group separately (i.e., Kenyan versus non-Kenyan groups). We defined country of origin as the place of birth of the caretaker and his/her parents’ place of birth.

### Ethical review

The protocol for conducting the HUS was reviewed and approved by the Scientific Steering Committee and the Ethical Review Committee of Kenya Medical Research Institute (KEMRI). It was also reviewed by the Centers for Disease Control and Prevention (CDC) and was determined to be non-research and exempt from Institutional Review Board (IRB) approval.

## Results

### General demographics

**Figure 1:**
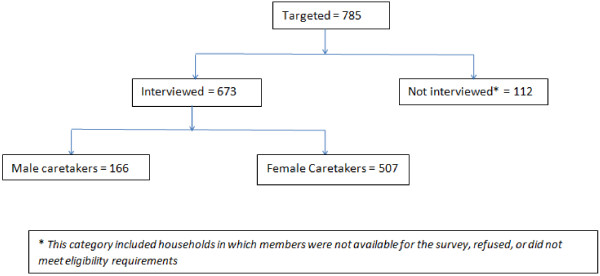
Number of households in different sample sizes and categories, (Eastleigh, July – August 2010).

We collected data from 673 (85.7%) households with 3,005 individuals (Figure 1). The median household size was 4 (range 1–19) people. Thirteen households (1.7%) refused to participate in the survey, 24 (3.1%) were ineligible (i.e., household that were randomly selected that turn out to be none residential or households that lived in the study area for less than 3 months or household where there was no caretaker that was above 18 years). There was no response in 68 (8.7%) households after the fourth attempt to find them at home. The primary caretakers were mostly women (507 [75.3%]) with a median age of 29 years (range 18–85 years). The predominant languages used in the households were Somali (75.2%), Kiswahili (11.9%), and Oromo (from Ethiopia) (6.0%). The majority of the caretakers (52.3%) reported Somalia as the country of birth, while 230 (39.1%) were born in Kenya and 50 (8.0%) in Ethiopia (Table 1). When caretakers were asked about highest level of education attained, 143 (21.2%) reported no education, 133 (19.8%) had only religious education, and 193 (28.7%) completed high school or higher levels of education. Majority of the caretakers (90.9%) reporting no education were female (Table 1).

**Table 1:** General household characteristics, by the gender of the caretaker (Eastleigh, July – August 2010)

**Background characteristics**		**Female**	**Male**	**Total**
		**507 (75.3%)**	**166 (24.7%)**	**673 (100%)**
Age (yrs)	Mean	32.7	32.6	32.65
	Median (Range)	29 (18–85)	29 (16–80)	29 (16–85)
Age groups*	<20	17 (4.6)	15 (8.2)	32 (5.5)
	20-35	327 (62.2)	90 (50.9)	417 (59.2)
	35-50	99 (21.1)	40 (27.2)	139 (22.7)
	50+	62 (11.8)	21 (13.7)	83 (12.3)
Language predominantly spoken in the household^$^	Somali	387 (76.5)	118 (71.1)	505 (75.2)
	Kiswahili	57 (11.3)	23 (13.9)	80 (11.9)
	Oromo	27 (5.3)	13 (7.8)	40 (6.O)
	English	3 (0.6)	3 (1.8)	6 (0.9)
	Others	32 (6.32)	9 (5.42)	41 (6.1)
Country of birth	Somalia	311 (61.3)	78 (47.0)	389 (52.3)
	Kenya	163 (32.1)	67 (40.1)	230 (39.1)
	Ethiopia	30 (5.9)	20 (12.0)	50 (8.0)
	Eritrea	1 (0.2)	1 (0.8)	2 (0.3)
	Djibouti	1 (0.2)	0 (0.0)	1 (0.2)
	Saudi Arabia	1 (0.2)	0 (0.0)	1 (0.0)
Highest level of education achieved^^^	No education	130 (25.6)	13 (7.8)	143 (21.2)
	Only religious education	116 (22.9)	17 (10.2)	133 (19.8)
	Less than primary school	39 (7.7)	7 (4.2)	46 (6.8)
	Primary school or equivalent	111 (21.9)	32 (19.3)	143 (21.2)
	Secondary school or equivalent	60 (11.8)	54 (32.5)	114 (16.9)
	Post secondary education	40 (7.9)	32 (19.3)	72 (10.7)
	Bachelors degree and above	1 (0.2)	6 (3.6)	7 (0.1)

*Two female caretakers did not know their age.

^*$*^*The language data for one caretaker are missing.*

^Fifteen caretakers only attended Madarasa, where the curriculum is different from Kenyan curriculum.

Note: All percentages are weighted.

### Water and sanitation

When surveyed about their sources of drinking water, 390 (57.9%) of the households reported obtaining their drinking water from a household tap, while 230 (34.2%) obtained their drinking water from either a public/shared tap or from a vendor. Few households (52 [7.7%]) reported buying bottled water for drinking. Only 219 (33.6%) of the households reported treating their drinking water by boiling or using commercial products. Three hundred fifty-three (50.3%) households had their own toilets, compared with 315 (48.2%) households that used communal/shared toilets. Four (1.3%) households reported using pit latrines.

Fundamental practices differed depending on the gender of the caretaker; households for which the caretakers were female were more likely to have a household tap as a source of drinking water than were households with a male caretaker (Unadjusted Odds Ratio [OR] =2.04; 95% Confidence Interval [CI] 1.28-3.26; p = 0.0027). Similarly, households for which caretakers were female were more likely to have household toilets than were households with male caretakers (OR = 1.98; 95% CI 1.23-3.20; P = 0.0052).

### Frequency of reported illness

Of the 3,005 participants, 566 (18.8%) reported at least one of the illnesses of interest. ARI was reported by 290 (9.7%), fever by 222 (7.4%), and diarrhea by 54 (1.8%) participants. Children under the age of 5 years had the highest frequency of all the syndromes: 17.1% (95% CI 12.2-21.9) had ARI, 10.0% (95% CI 6.2-13.8) fever, and 6.9% (3.8-10.0) diarrhea (Table 2).

**Table 2:** Frequency of reported illnesses among survey respondents, by age category (Eastleigh, July – August 2010)

**Age category (yrs)**	**No. of survey respondents**	**% reported ARI in past year (CI)**	**% reported diarrhea in past 30 days (CI)**	**% reported fever in past 2 weeks (CI)**
≤4	368	17.1 (12.2-21.9)	6.9 (3.8-10.0)	10.0 (6.2-13.8)
5-17	835	9.2 (6.6-11.7)	1.2 (0.4-2.0)	7.7 (5.1-10.3)
18-50	1648	9.9 (8–11.7)	1 (0.4-1.6)	7.2 (5.7-8.7)
>50	147	11.8 (6.4-17.2)	0.4 (0–1.2)	11.1 (4.4-17.9)
Total	2998	10.7 (9.3-12.1)	1.8 (1.2-2.3)	7.9 (6.7-9.2)

- CI = 95% confidence interval.

- Seven (7) people did know their age, hence were not included in this table.

- ARI: Acute respiratory illness, including pneumonia.

- Note: All percentages are weighted.

Among all age groups, 29 (7.5% [95% CI 4.3-10.7]) hospitalizations occurred among those who sought medical care within the syndrome-specific time frames. The illness with highest proportion resulting in hospitalization was diarrhea (16.7%), followed by ARI (7.5%) and febrile illness (3.8%) (Table 3). Eight (0.3%) deaths were reported in the 12 months preceding the survey in the households surveyed. Only one death was related to the syndromes surveyed: in a 2-year-old child who was reported to have had pneumonia.

**Table 3:** Healthcare utilization for episodes of acute illnesses (Eastleigh, July – August 2010)

**Variable**	**Total (N = 434)**	**% (95% CI)**	**Fever (N = 129)**	**% (95% CI)**	**ARI (N = 262)**	**% (95% CI)**	**Diarrhea (N = 49)**	**% (95% CI)**
Care-seeking behavior								
Sought care	330	77.0 (72.2-81.9)	85	70.6 (60.3 - 80.9)	208	79.9 (74.1 - 85.8)	37	75.8 (61.0 - 90.5)
Did not seek care	99	23.0 (18.1-27.8)	36	29.4 (19.1 - 39.7)	52	20.1 (14.2 - 25.9)	11	24.2 (9.5 - 39.0)
Reasons for not seeking care								
Was not sick enough	33	34.6 (23.0-46.2)	15	43.4 (23.0 - 63.7)	15	31.1 (15.1 - 47.1)	3	25.5 (0.0 - 54.5)
Was getting better on own	11	8.7 (2.9-14.6)	3	5.7 (0.0 - 12.3)	6	8.8 (0.7 - 16.9)	2	17.8 (0.0 - 43.6)
Too expensive	39	44.8 (32.7-57.0)	13	42.6 (21.2 - 64.0)	22	45.9 (29.6 - 62.2)	4	46.2 (10.5 - 81.9)
No one to take care of the children/house while gone	3	3.2 (0.0-7.5)	0		3	5.9 (0.0 - 13.4)	0	
Site of care*								
Private health care provider	174	59.8 (52.5 -67.1)	42	59.2 (44.3 - 74.0)	111	60.3 (51.2 - 69.4)	21	58.3 (37.2 - 79.4)
Pharmacy/Chemist/Drug seller	55	18.9 (8.6 - 29.2)	15	21.1 (0.5 - 41.8)	35	19.0 (6.0 - 32.0)	5	13.9 (0.0 - 44.2)
Government facility	38	13.1 (2.3 - 23.8)	9	12.7 (0.0 - 34.4)	22	12.0 (0.0 - 25.5)	7	19.4 (0.0 - 48.8)
Individual^†^	24	8.2 (0.0 - 19.3)	5	7.0 (0.0 - 29.5)	16	8.7 (0.0 - 22.5)	3	8.3 (0.0 - 39.6)
Medications used								
Antibiotics	176	40.0 (34.3-45.7)	24	17.3 (9.6 - 25.0)	139	52.0 (44.5 - 59.4)	13	25.5 (11.3 - 39.8)
Painkiller	173	36.5 (31.0-41.9)	72	60.1 (48.9 - 71.4)	93	29.5 (23.3 - 35.8)	8	18.5 (4.4 - 32.7)
Anti-malarial	49	9.1 (6.2-12.1)	39	28.8 (19.1 - 38.4)	10	2.4 (0.7 - 4.1)	0	
ORS^‡^	21	5.2 (2.6-7.7)	0		0		21	47.9 (31.0 - 64.8)
Hospitalization	29	7.5 (4.3-10.7)	6	3.8 (0.0 - 8.6)	16	7.5 (3.5 - 11.5)	7	16.7 (3.9 - 29.5)
Diagnostics done								
X-ray	46	21.4 (13.7-29.2)			46	21.4 (13.7 - 29.2)		
Stool microscopy	24	58.1 (41.4-74.9)					24	58.1 (41.4 - 74.9)
Blood smear for malaria	62	42.2 (30.7-53.6)	62	42.2 (30.7 - 53.6)				
Malaria parasites present	42	27.7 (17.9-37.5)	42	27.7 (17.9 - 37.5)				

*The name of the site of care for each respondent was asked, and only those who knew the names of the site were included in this analysis.

† Individuals included relatives, friends, religious leaders, traditional healers, and health volunteer.

‡ORS – Oral rehydration solution.

Note: All percentages are weighted.

There were no differences in illness frequency between males and females.

### Overview of health care utilization

**Figure 2:**
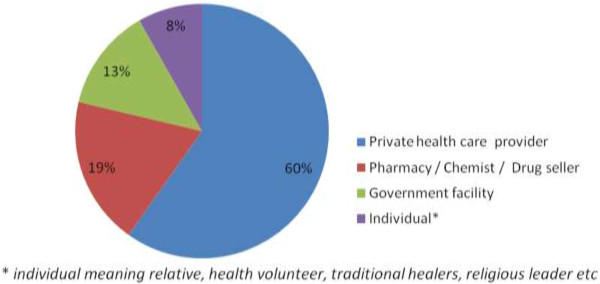
Percentage of individuals seeking health care at different institutions (Eastleigh, July – August 2010).

Of the 566 participants with a history of one of the illnesses, 434 (76.7%) were asked questions relating to health care utilization, and 330 (77.0%) reported seeking some kind of health care. Among those who sought care, 199 (48.3%) were advised by a third party to do so, in most cases by a relative or a friend. Most of those seeking health care went to private health care providers [n = 174 (59.8%)], followed by pharmacies [n = 55 (18.9%)]. Only 38 (13.1%) ill residents sought care at government-run facilities; 24 (8.2%) sought care from a relative, religious leader, or a health volunteer (Figure 2).

We asked the 434 individuals who responded to the health care utilization questions about the type of investigations and treatment they received in association with each of the illnesses of interest to determine if respondents received appropriate health care services. Those who reported having fever were asked if a blood smear was done for malaria; those who met the case definition of ARI were asked if a chest x-ray was done; and those who reported diarrhea were asked if a stool specimen was collected and an examination done.

Sixty-two (42.2%) blood smears were done for those who reported fever to screen for malaria; malaria parasites were reported in 42 (67.7%) of those blood smears. Chest x-rays were done for 46 (21.4%) of the respondents with ARI symptoms. Stool examinations were done for 24 (58.1%) persons who had diarrhea in the previous 30 days.

Antibiotics were used to treat 139 (52.0%) of the reported ARI cases, 13 (25.5%) diarrheal diseases, and 24 (17.3%) of the febrile illness cases. Antimalarial drugs were given to 39 (28.8%) of those with febrile illness and 10 (2.4%) with ARI. Of those who reported diarrheal disease, 21 (47.9%) received oral rehydration salts (Table 3).

### Factors influencing health care utilization

Although some form of health care was sought in the majority of cases, 99 (23.0%) patients across the syndromes reported that they did not seek care. The highest percentage of those not seeking care were those with acute febrile illness (29.4%) compared with those with ARI (20.1%) and diarrhea (24.2%) (p > 0.05) (Table 3). The primary reasons for not seeking care were the care was too expensive (n = 39 [44.8%]), the person was not sick enough (n = 33 [34.6%]), the person was getting better on his/her own (n = 11 [8.7%]), and no one was available to take care of the children and the house while the caretaker was gone (n = 3 [3.2%]) (Table 3).

Children under the age of 5 years were more likely to receive health care services for diarrheal diseases than were persons older than 5 years (94.1% vs 56.7%, p = 0.009). Similarly, children under 5 years were more likely to receive medication to treat diarrheal disease than were older participants (94.4% vs 64.0% p = 0.019). Health care utilization for febrile illnesses was similar in all age categories.

Participants were more likely to use health care services if a family member cared for them during the illness; however, the association was marginally significant (OR 1.77; 95% CI 0.98 – 3.19; p = 0.057).

We examined whether country of origin influenced health care utilization among participants. We found that among Kenyans no factors significantly influenced health care seeking behavior. In contrast, among the non-Kenyan group, many factors seemed to influence health care seeking behavior, including father’s country of origin, severity of illness, recommendation to access health care, SES, and educational level. Non-Kenyan participants were more likely to seek health care services if the father’s origin was in Ethiopia compared to if the father’s origin was in Somalia and if the illness was severe but this was not statistically significant (Table 4) and were more likely to seek care if they were advised to seek medical attention (OR 2.83; 95% CI 1.43-5.60; p = 0.003). Non-Kenyans in the middle SES group were also significantly more likely to seek health care services (OR 3.04; 95% CI 1.39-6.63; p = 0.005). When the data were fitted into a multivariate model where variables with p value <0.1 were included, three variables were significantly associated with health care seeking behavior: recommendation by a third party to seek health care services, father’s origin in Ethiopia and being in the middle SES category. Health care utilization among non-Kenyan participants did not differ significantly based on the caretaker’s age, gender, language, household size, and the age of the sick person.

**Table 4:** Factors associated with healthcare utilization for non-Kenyan respondents (Eastleigh, July – August 2010)

**Variable**	**Category**	**Total**	**Seeking care n (%)**	**Not seeking care n (%)**	**Unadjusted odds ratio (95% CI)**	**P value**	**G_pvalue**
Language predominantly spoken in household	Somali	277	212 (75.5)	65 (24.5)	1.22 (0.33 - 4.54)	0.7695	
	Oromo	16	12 (71.6)	4 (28.4)			
	Other	14	12 (91.8)	2 (8.2)	4.42 (0.60 - 32.62)	0.1452	0.2586
Country of origin*	Somalia	267	201 (73.6)	66 (26.4)			
	Ethiopia	43	37 (89.9)	6 (10.1)	3.20 (1.15 - 8.94)	0.0265	0.0265
Advised to go to the hospital	Yes	145	124 (85.5)	21 (14.5)	2.83 (1.43 - 5.60)	0.0028	0.0028
	No	163	112 (67.5)	51 (32.5)			
Gender of household member	Male	58	43 (71.8)	15 (28.2)			
	Female	252	195 (77.3)	57 (22.7)	1.34 (0.61 - 2.96)	0.4689	0.4689
Age of household member	<5years	71	58 (79.5)	13 (20.5)	1.27 (0.57 - 2.82)	0.563	0.563
	≥5years	239	180 (75.3)	59 (24.7)			
Caretaker’s Education	No school or religious education	82	64 (74.5)	18 (25.5)			
	Religious education	70	52 (69.9)	18 (30.1)	0.80 (0.33 - 1.95)	0.6177	
	Only primary school or less	85	64 (81.2)	21 (18.8)	1.48 (0.59 - 3.68)	0.4005	0.5859
	Secondary school or higher	73	58 (78.1)	15 (21.9)	1.22 (0.49 - 3.04)	0.666	
Household size	1- < 3	49	38 (70.1)	11 (29.9)			
	3- < 5	86	67 (79.3)	19 (20.7)	1.63 (0.59 - 4.53)	0.3462	
	5- < 8	96	71 (77)	25 (23)	1.43 (0.54 - 3.78)	0.4743	
	≥8	79	62 (75.7)	17 (24.3)	1.33 (0.46 - 3.80)	0.5972	0.8213
Who cared for the person during the illness	No one/cared for self	87	61 (68.7)	26 (31.3)			
	Other family member	219	173 (78.6)	46 (21.4)	1.67 (0.84 - 3.34)	0.1459	0.1459
Social Economic Status	Higher	107	73 (65.3)	34 (34.7)			
	Middle	125	104 (85.1)	21 (14.9)	3.04 (1.39 - 6.63)	0.0052	
	Lower	70	53 (75.4)	17 (24.6)	1.63 (0.74 - 3.57)	0.2251	0.0199
Severity	Severe	42	37 (88.1)	5 (11.9)			
	Mild	261	196 (75.1)	65 (24.9)	2.45 (0.93 - 6.51)	0.08583	0.08583

*The country of origin here refers to the country of origin of the household caretaker inferred from his/her place of birth and his/her parent’s place of birth.

Note: All percentages are weighted.

### Cost of health care services

The median cost per visit for fever, diarrheal disease, and ARI varied depending on the site of care. While the median cost at government facilities was Ksh. 50 (USD 0.58) (Range: Ksh. 0 – Ksh. 26,000), the median cost increased to Ksh. 2,260 (USD 26.28) (Range: Ksh. 20 – Ksh. 43,500) in private facilities, regardless of the syndrome. For persons who sought care directly from a pharmacy, the median expenditure was Ksh. 1,500 (USD 17.44) (Range: Ksh. 50– Ksh. 11,000).

## Discussion

Our study found that more than 78.7% of residents of Eastleigh who sought some form of health care either went to privately owned health care facilities or bought medications without consulting a clinician. Overall, participants in our study preferred private clinics over government-run health care facilities. For example, although the local city council clinic has affordable prices (i.e., Ksh. 20 or USD 0.23) and is within walking distance to the survey participants, only 11.2% of those with acute illnesses sought health care services there. Another 1.9% participants indicated that they accessed health care services in government facilities outside Eastleigh. In total, 13.1% residents seeking any type of health care went to a government facility. Clearly, despite their accessibility and affordability, government facilities are not being used by most individuals in this population.

Studies have shown fear of government authorities and perceived low quality of health care services as reasons why urban refugees tend to avoid government facilities [21]. Although our study did not specifically ask about refugee status, we estimate that over 50% of participants were possibly refugees considering place of birth of the participant and the places of birth of both parents, which were part of the questions we asked. By law in Kenya, refugees are not restricted from accessing health care services from government-run facilities. However, other studies have reported that refugees may have anxiety about receiving services from government officials because of policies requiring refugees to remain in camps; such concerns may drive refugees to seek care at informal, private facilities or to self-treat [2]. As more and more residents turn to private health care providers and pharmacies, government regulations and oversight will need to keep in step. Our study did not collect information on the quality of care at the privately owned clinics, but other studies have shown that in many developing countries, including Kenya, most of the people selling medicines at these unregulated private facilities are not trained or licensed pharmacists or medical practitioners. As such, there is a higher risk that the unregulated private facilities may be dispensing incorrect drugs or inappropriate courses of treatment based on a person’s ability to pay [22,23].

As the number of refugees residing in urban areas increases globally, UNHCR and its partners in health need to understand the health profile and needs of this growing population. One major challenge, however, is the difficulty of identifying refugees and establishing their population size in these urban areas. The lack of denominator data hampers planning. Because urban refugees are hidden, it is difficult to set up health information systems [1]. Studies in other parts of the world have reported that migrants and refugees in urban areas usually have poor access to public health care services [24]. People moving into urban areas opt to seek care at unregulated clinics or conduct unsupervised self treatment [25].

Our study found that more than three-quarters of participants sought some form of health care when sick. However, compared with other populations in Nairobi or rural populations elsewhere in Kenya, the proportion accessing health care services in our study was lower for all illnesses of interest [20,26]. This relatively low level of health services utilization in this area with high concentration of urban refugees is supported by results of other studies in similar populations, which have indicated that displaced refugee communities have poorer or suboptimal access to health care compared with the local communities [27-29].

When we looked at factors that affect health care-seeking behavior, residents of Eastleigh North Section II were more likely to seek health care services when advised to do so by others. This finding is also supported by other studies that established similar correlations between seeking care and being advised to seek care [30]. This finding emphasizes the importance of interventions by clinicians, family members, community health workers, and others in the community. We also found that health care-seeking behavior significantly increases when there is a family member caring for the ill person.

In our study we found that the primary reason why some respondents did not seek health care was because of affordability issues, those respondents said care was expensive. The fact that any form of payment made for health services deters patients from seeking care is proven in many studies. This interferes with equity in service provision [31].

### Limitations

The area we surveyed is reputed to have a large percentage of Somali and Ethiopian people who consider themselves urban refugees, but are residing in Kenya without any legal documentation of their immigration status. However, no studies have been done to specifically ascertain the numbers and the immigration status of people living in Eastleigh. During the time of this study, the immigration status of individuals living in Eastleigh was of special concern to the local communities and the media. Some media outlets and political leaders were accusing Eastleigh residents of money laundering and questioning their immigration status. In this context, we believed Eastleigh residents may have been suspicious of anyone asking about money or nationality. To minimize this suspicion, we designed the survey instrument to exclude any direct questions about immigration status, country of origin or income. Instead, respondents were asked to name the caretaker’s and his/her parents’ place of birth, to identify some of the migratory patterns of this population. Primary language of the household was also assessed to support the cultural origins of the population. These types of indirect questions have limitations in conclusively identifying the refugee status of those surveyed, but help to describe the sample population’s basic demographics. Similarly, by using the Principal Component Analysis (instead of direct questions about income), we were able to classify the participating households into socioeconomic strata. This approach may represent limitations in conclusively determining a household’s SES.

A second area of limitation is data collection through self-reporting. Data on illnesses of interest were based on participants’ self-report, without confirmation by a health care professional. This may have led to misclassifications of illness, especially for pneumonia and fever. For example, while the assessment of diarrhea is fairly straightforward, the assessment of fever is more subjective, especially when thermometers are not used. Such nonspecific measures have the potential to bias estimates of prevalence upward. Self-reported data could also blur the relationship between valid syndromes and associated health care-seeking behaviors. Similarly, we relied on self reporting for past illnesses, particularly with regard to the episodes of pneumonia that occurred in the 12 months before the survey. This approach may have introduced a recall bias into the survey.

In our study, 14.3% of the initially selected households were not interviewed. These were households that either refused to participate in the study or were not present for interview after the fourth visit. While we think the characteristics of the non-responders are similar to those of the responders, we were not able to assess and confirm this.

The cross-sectional survey evaluated the prevalence of syndromes of diarrhea and fever at a specific point in time; seasonal variations could therefore have played a key role in the prevalence observed.

## Conclusion

The findings from this survey have implications for both health care delivery and public health interventions. Of particular concern is the large proportion of Eastleigh North Section II residents who seek care at private providers or buy drugs without consultation with a clinician. Informal, private health care providers and pharmacies pose significant health risks because there is no oversight or regulation of such facilities. Without any oversight framework, there is increased risk that life-threatening illnesses will not receive adequate treatment in these facilities. Likewise, unregulated administration of antibacterial drugs may contribute to the development of drug resistance. The informal, private clinics and pharmacies also represent a gap in national surveillance projects, since most of these facilities do not report communicable diseases through the national reporting system. Such a gap in national health data will likely reduce the government’s ability to manage public health concerns and respond to epidemics in a timely manner. Consequently, the findings of our study indicate the value of including informal, private health facilities in systematic disease surveillance in Nairobi. The information from this survey will provide a foundation for establishing, interpreting, and evaluating future surveillance activities. Further studies will be necessary to investigate the reasons many immigrant communities avoid government-run facilities for their health care needs.

## Abbreviations

ARI: Acute respiratory illness; CDC: US Centers for Disease Control and Prevention; CI: Confidence interval; HUS: Health care utilization survey; IRB: Institutional Review Board; KEMRI: Kenya Medical Research Institute; Ksh.: Kenya Shillings; OR: Odds Ratio; PCA: Principal Component Analysis; PPS: Probability Proportional to Size; SES: Socioeconomic Status; UNHCR: United Nations High Commissioner for Refugees; USD: US Dollar; WHO: World Health Organization.

## Competing interests

The authors declare that they have no competing interest.

## Authors’ contributions

AHM, RBE,WD, HB and JA contributed to the conception and design of the study, acquisition, analysis and interpretation of data, and have been involved in drafting the manuscript and revising it critically for important intellectual content . RN, EA and IN contributed in project implementation, data collections, entry, cleaning and analysis, and drafting of the manuscript. WS and RFB contributed in study implementation, drafting of manuscript and revising it critically for important intellectual content. All authors read and approved the final manuscript.

## Pre-publication history

The pre-publication history for this paper can be accessed here:

http://www.biomedcentral.com/1472-6963/14/200/prepub
